# Patient-Reported Experiences in Voxelotor-Treated Children and Adults with Sickle Cell Disease: A Semistructured Interview Study

**DOI:** 10.1155/2023/7533111

**Published:** 2023-01-28

**Authors:** Clark Brown, Modupe Idowu, Richard Drachtman, Anne Beaubrun, Irene Agodoa, Andy Nguyen, Kelly Lipman, Olga Moshkovich, Ryan Murphy, M. Alex Bellenger, Wally Smith

**Affiliations:** ^1^Aflac Cancer & Blood Disorders Center of Children's Healthcare of Atlanta and Department of Pediatrics, Emory University, Atlanta, GA, USA; ^2^UT Physicians Comprehensive Sickle Cell Center, The University of Texas Health Science Center at Houston, Houston, TX, USA; ^3^Rutgers Cancer Institute of New Jersey, New Brunswick, NJ, USA; ^4^Global Blood Therapeutics, Inc., South San Francisco, CA, USA; ^5^ICON plc, Raleigh, NC, USA; ^6^VCU Adult Sickle Cell Medical Home, Division of General Internal Medicine, Virginia Commonwealth University, Richmond, Virginia, USA

## Abstract

**Objective:**

Voxelotor is a first-in-class sickle hemoglobin–polymerization inhibitor that was approved in 2019 by the US Food and Drug Administration for treatment of patients with sickle cell disease (SCD) aged ≥12 years; in 2021, the approval was extended to children with SCD aged 4 to 11 years. Additionally, both the Ministry of Health and Prevention for the United Arab Emirates and the European Commission granted marketing authorization for voxelotor in September 2021 and February 2022, respectively, for treatment of SCD in adults and pediatric patients aged ≥12 years. Thus, additional information on the patient experience with voxelotor would be useful for patients, caregivers, and healthcare professionals alike. The purpose of this study was to conduct semistructured interviews in an effort to understand the experiences and perspectives of voxelotor-treated patients with SCD.

**Methods:**

One-time semistructured interviews with adults, adolescents, and children with SCD and their primary caregivers were conducted in the United States. Twenty-three adults and adolescents were recruited across 4 clinical sites, and 10 children-caregiver dyads were recruited from a single site. The interview was designed to elicit patient perspectives on symptomatic changes with voxelotor and the impact of treatment on patients' perceived health-related quality of life. Individual interview transcripts were analyzed using a thematic analytic approach, and concept saturation was assessed in both cohorts.

**Results:**

Most patients reported improvements in their SCD symptoms with voxelotor treatment, specifically regarding pain crises, jaundice, and fatigue. Almost all patients experienced improvements in self-reported health-related quality of life with voxelotor treatment.

**Conclusions:**

This study provides patient and caregiver perspectives on the symptomatic benefits of voxelotor treatment. These findings not only highlight the benefits of voxelotor treatment in improving symptoms and increasing health-related quality of life across the entire SCD population but also can inform further research on SCD-specific patient-reported outcomes.

## 1. Introduction

Sickle cell disease (SCD) is the most common inherited blood disorder in the United States, affecting approximately 100,000 Americans [1, 2]. Globally, an estimated 4.4 million people have SCD, and an additional 43 million have sickle cell trait [3]. Approximately 80% of SCD cases are believed to occur in sub-Saharan Africa, but SCD is also widely prevalent in the Mediterranean, the Middle East, and India [4, 5].

SCD results from mutations in the beta-globin gene that lead to formation of sickle hemoglobin (HbS). When deoxygenated, HbS rapidly polymerizes and deforms red blood cells (RBCs) into a rigid sickle shape [4, 6]. Repetitive episodes of HbS polymerization and RBC sickling damage the cell membrane, causing RBCs to become irreversibly sickled and prone to intravascular hemolysis [6]. Sickled RBCs can cause recurrent vasoocclusion, leading to acute and chronic complications due to repeated tissue ischemia and inflammation [6]. HbS polymerization, vasoocclusion, and hemolytic anemia precipitate a cascade of pathologic events that lead to a wide variety of organ dysfunction [5, 6]. Chronic hemolysis and subsequent chronic anemia and vascular dysfunction are associated with increased risk of developing pulmonary arterial hypertension, priapism, and leg ulcers [7].

Signs and symptoms of SCD usually appear in infancy or early childhood and persist throughout adulthood [3, 8, 9]. Patients with SCD experience a range of symptoms, including pain, fatigue, jaundice, and respiratory problems, as well as chronic complications such as pulmonary hypertension and multisystem end-organ damage [10]. Pain can present as chronic pain, defined as pain that is present on most days for at least 6 months, or as acute vasoocclusive crises (VOCs) [10, 11]. VOCs are recurrent, painful events caused by sickled RBCs that block blood vessels, impair oxygen delivery, and cause subsequent infarction and infarction-reperfusion injury [4, 6, 12]. VOCs are unpredictable in nature and are the main reason for SCD-related hospitalizations [4, 12]. Chronic pain is progressive, with onset during childhood and increased prevalence with age, and is often associated with functional disability and psychiatric illnesses [11, 13]. Fatigue is also a commonly reported symptom that is associated with anxiety, depression, stress, and lower quality of life [14]. Additionally, hemolytic anemia causes jaundice and has been implicated in the pathogenesis of leg ulcers, a painful and often debilitating chronic complication of SCD [7, 15, 16].

Because of its chronic nature and the early onset and severity of symptoms, SCD negatively impacts the health-related quality of life (HRQOL) of both adults and children with SCD [4, 17]. Thus, HRQOL has become an emerging interest in SCD research. Measures of HRQOL have been used by clinicians to identify prognostic factors of disease progression and to determine the clinical effectiveness of an intervention [17].

There are several FDA-approved drugs and various disease-modifying and curative therapeutic approaches for the treatment of SCD, but many limitations exist. In particular, transfusion therapy may be associated with various complications or inconveniences. Complications include alloimmunization, iron overload, and hyperviscosity [18]. Inconveniences include the time-consuming clinic visits and need to regularly monitor patients for transfusion-related side effects, which add to the substantial disease burden for patients and their families [19].

Among the FDA-approved disease-modifying agents for the treatment of sickle cell anemia in adults and children, hydroxyurea is by far the oldest and most widely recommended (especially for homozygous HbS and heterozygous HbS/*β*^0^ thalassemia). Unfortunately, prescription of and compliance with hydroxyurea treatment are not uniform, and adherence is low [20–22]. Physicians may be unfamiliar with the proper prescribing of hydroxyurea in the treatment of SCD or have concerns about patient compliance [23]. But more importantly, patients and families may be concerned about potential side effects of hydroxyurea, which are noted in the product insert, anecdotally experienced, or informally suspected yet strongly believed by patients and prescribing physicians [24, 25]. Hydroxyurea use is therefore associated with high rates of treatment discontinuation and poor medication adherence [24, 26].

Currently, hematopoietic stem cell transplantation remains the only cure for SCD [8]. Despite its potential, finding an unaffected matched donor can be difficult, and the procedure has serious risks, including death [6, 8]. Recently, gene therapy has been used as an experimental alternative cure, but the potential and safety of this therapy are currently unknown [27, 28].

As a HbS polymerization inhibitor, voxelotor modulates the affinity of hemoglobin for oxygen and has the potential to be a disease-modifying treatment for SCD. By binding to and stabilizing HbS in an oxygenated state, voxelotor directly inhibits HbS polymerization, prevents repeated RBC sickling, and improves RBC health [29, 30]. Inhibition of HbS polymerization, the root cause of SCD pathology, has the potential to prevent RBC hemolysis, anemia, and other downstream complications of SCD.

Voxelotor is a first-in-class HbS polymerization inhibitor that was approved in the United States in 2019 for patients with SCD aged ≥12 years and in 2021 for children aged 4 to 11 years [31]. Marketing authorization for voxelotor was also granted in the United Arab Emirates (September 2021), the European Union (February 2022), Great Britain and Oman (July 2022), and Kuwait (September 2022) for the treatment of SCD in patients aged ≥12 years [32]. Approval was based on results from the pivotal phase 3 HOPE trial in which treatment with voxelotor for 24 weeks demonstrated robust increases in hemoglobin levels, reduced hemolysis, and improved Clinical Global Impression of Change ratings compared with placebo, with improvements maintained at 72 weeks [33]. Additional real-world use and patient perspectives and insights are needed to further appreciate the effects of voxelotor on the patient experience. Generating qualitative data using both patient and caregiver insights can lead to a better understanding of the meaningful treatment benefits in this patient population and help integrate the patient voice into the assessment of voxelotor. Therefore, the objective of this study was to collect semistructured interview data to understand the experiences and perspectives of patients with SCD being treated with voxelotor.

## 2. Materials and Methods

### 2.1. Study Design

This study employed one-time, semi-structured telephone interviews with adults (aged ≥18 years), adolescents (aged 12-17 years), and children (aged 4-11 years) and their primary caregivers. The study protocol and materials were reviewed and approved by Western IRB (Independent Review Board; http://www.wcgclinical.com) in the United States (protocol 3507-0047, approval date August 20, 2020). Participants were recruited through clinical sites with a sickle cell population, and each of these sites also received local IRB approval. Once participants were determined to be eligible and willing to participate in the study, interviewers scheduled a one-time, one-on-one telephone or video conference interview.

### 2.2. Participants

Adult and adolescent patients with SCD were recruited from 4 clinical sites, and children with SCD were enrolled from 1 site, all in the United States. The sampling frame simulated that of a purely qualitative study. Key inclusion criteria were the following: SCD diagnosis, age of at least 4 years, and currently taking voxelotor for SCD treatment (for ≥4 weeks for those aged ≥12 years). Caregivers of children were required to be aged 18 years or older. Participants and caregivers provided informed consent or assent.

### 2.3. Interview Development and Procedures

The interview questions and guide were developed by ICON plc in consultation with the study sponsor, Global Blood Therapeutics, Inc. ICON plc piloted the interview guide internally and refined it prior to conducting interviews with study participants.

All interviews were conducted in English, via telephone or video conference, from April 2021 to September 2021. Interviewers were trained in qualitative research methods, the study-specific aims, and interview administration procedures. The interviews proceeded according to the appropriate approved semistructured interview guide and lasted approximately 60 minutes (30 minutes for children). Some interview questions required quantitative responses; other questions required qualitative or descriptive responses. All interviews were audio recorded. Audio recordings of the interviews were transcribed verbatim and reviewed for accuracy. All identifiable information was removed from the transcripts to ensure confidentiality.

For each SCD sign or symptom, patients were asked to rate the severity on a scale of 0 to 10, with 0 being not at all severe and 10 being extremely severe. Patients were also asked to rate the symptom's impact on their overall quality of life on a scale of 0 to 10, with 0 being not at all impactful and 10 being extremely impactful. Patients were asked to rank their HRQOL on a scale of 0 to 10, with 0 being very poor and 10 being very good.

### 2.4. Data Analysis

Sample composition data and quantitative interview responses were analyzed using simple descriptive statistics. Qualitative transcript responses were analyzed with MAXQDA 2020 qualitative analysis software (http://www.maxqda.com). Analysis of concept elicitation interview data was conducted using a thematic analytic approach, which is a rigorous and transparent method that is well suited to qualitative research [34]. An initial codebook was developed based on concepts and themes (e.g., symptoms, impacts) included in the interview guide. Analysts assigned thematic codes to sections of text (e.g., sentences, lines, paragraphs) and generated new codes as appropriate. These codes were then organized into categories (e.g., symptoms, impacts), which were defined by the content of the coded sections of text and supported by example quotations. At an early stage, approximately 10% of the transcripts were independently double-coded by two analysts to ensure consistency in approach. Additionally, concept saturation—the point at which new concepts no longer emerge through further data collection—was assessed in both cohorts through a saturation grid documenting conceptual coverage [35].

## 3. Results

### 3.1. Participants

A total of 23 adults and adolescents and 10 child-caregiver dyads were interviewed. All children (*n* = 10) had received voxelotor through their participation in a clinical trial because voxelotor was not yet approved for pediatric patients aged <12 years at the time of this study. At the time of the interview, adults and adolescents had been taking voxelotor for an average of 0.7 years (range 0.25-1.7 years), and children had been taking voxelotor for an average of 1 year (0.25-2 years). Most patients (adults, adolescents, and children) and the children's caregivers were female and of Black or African American descent (Tables 1 and 2).

### 3.2. Thematic Concept Saturation

When assessing data saturation across adult and adolescent interviews, 30 unique concepts were identified in the 23 interviews. A total of 90% of concepts were identified by the tenth interview, and all were reported by the seventeenth interview. In the caregiver interviews, 21 unique concepts were identified across the 10 interviews, with 62% of concepts identified by the second interview and all concepts reported by the third interview. In the interviews with children, 18 unique concepts were identified across the 10 interviews, with 56% of concepts identified by the second interview and all concepts identified by the tenth interview.

### 3.3. Baseline Symptoms before Voxelotor

Patients and caregivers were asked to recall which SCD symptoms they experienced before initiating voxelotor. In adults and adolescents, the most frequently reported symptoms were pain crises (23/23; 100%), jaundice (19/23; 83%), and fatigue (19/23; 83%). The most common symptoms caregivers reported for their child included fatigue (8/10; 80%), pain crises (7/10; 70%), jaundice (5/10; 50%), respiratory illnesses (3/10; 30%), and chronic or breakthrough pain (3/10; 30%). For children reporting their own symptoms, pain (6/10; 60%), pain crises (5/10; 50%), and fatigue (4/10; 40%) were the most common.

### 3.4. Treatment Expectations

Adults, adolescents, and caregivers were asked about their hopes for treatment when starting voxelotor. The most-reported hopes in adults and adolescents were reduced symptoms (12/23; 52%), including pain crises, unspecified pain, fatigue, and jaundice; fewer hospital visits (5/23; 22%); improved quality of life (5/23; 22%); and supplementation or replacement of previous treatment (3/23; 13%). The most-reported hopes of caregivers were increased hemoglobin levels (5/10; 50%), reduction or elimination of pain crises (5/10; 50%), and improved quality of life for their child (3/10; 30%). Children were asked whether they were aware that they were taking a different or new medication for their SCD. Six children said they were aware of a change to their medication, and 4 reported that they were not.

### 3.5. Symptom Changes with Voxelotor Treatment

Participants were asked to describe changes in the most commonly reported symptoms (pain crises, jaundice, and fatigue) since starting voxelotor treatment. The percentages of interviewees who reported improvements in each symptom category are summarized in Table 3.

#### 3.5.1. Pain Crises

*(1) Pain Crises before Voxelotor*. All adults and adolescents (23/23; 100%) reported pain crises as a symptom of their SCD before starting voxelotor treatment.

“I would describe my pain would be like if someone was just like, I don't know, like hammering my back or something like that […] And then when it got to its worst it was like someone was really hammering on my back or just it was really intense.” (22-year-old patient with SCD)

Most caregivers (7/10; 70%) reported that their child experienced pain crises before starting voxelotor. Caregivers reported several notable impacts on their child's day-to-day life when pain crises occurred, including missing school (5/7; 71%), affected mental well-being (4/7; 57%), and lack of mobility (3/7; 43%).

“Sometimes it's the legs, [they're] not able to walk with the legs. Sometimes it's the arms, [they're] not able to raise them, sometimes just one leg, other times it's the chest.” (Caregiver of 10-year-old patient with SCD)

Five children (5/10; 50%) self-reported pain crises before voxelotor treatment.

“Sickle cell made my body feel terrible, like I would die. […] it made my body feel like I was going to die because the pain was so bad. Sometimes I couldn't even breathe.” (11-year-old patient with SCD)

*(2) Improvement in Pain Crises with Voxelotor*. Almost all of the adults and adolescents reported a positive change in their pain crises after treatment with voxelotor (Table 3), reporting reductions in severity (10/21; 48%), frequency (7/21; 33%), experiencing pain crises at all (5/21; 24%), and hospitalizations (3/21; 14%). The time until patients had a noticeable change in pain crises with voxelotor treatment varied widely: 1 to 2 months into treatment (3/21; 14%), 2 to 3 months (1/21; 5%), approximately 8 months (1/21; 5%), a little over 1 year (2/21; 10%), and during their first pain crisis (2/21; 10%). The average severity rating of pain crises decreased from 9.1 to 6.6, and the impact of pain crises on quality-of-life rating decreased from 7.7 to 4.9.

“I haven't really had any pain crises, and when I do they're always minor and it's never gotten to the point where I need to go to the hospital. And now, with the pain crises that I have had since taking it, instead of using something like oxycodone, I can [take] an ibuprofen and it'll go away fairly quickly. But they don't happen often. Like not as often as they used to be.” (22-year-old patient with SCD)

The majority of the caregivers reported improvements in their child's pain crises as a result of treatment with voxelotor, including decreases in severity, duration, and frequency of pain crises, and most of the children reported improvement in their pain crises after starting voxelotor (Table 3). No patients reported a worsening of their pain after starting voxelotor.

“…[they do] have pain crisis here and there, but it's not that really severe to the point that we can't control it.” (Caregiver of 9-year-old patient with SCD)

#### 3.5.2. Jaundice

*(1) Jaundice before Voxelotor*. Jaundice was reported as a symptom of SCD by 83% of adults and adolescents (19/23) and by 50% of caregivers (5/10) before initiating voxelotor.

“My eyes will get yellow if I knew I was going into a crisis. It was kind of my telltale sign that I was probably going to get admitted.” (22-year-old patient with SCD)

“It was really mild. It's really just most people probably wouldn't even notice it, but I noticed, […] how you're looking at [them] and [they're] talking to you and you're looking at [them], what is wrong with your eyes?” (Caregiver of 9-year-old patient with SCD)

*(2) Improvement in Jaundice with Voxelotor*. Jaundice was improved or resolved in almost all of the adults and adolescents who reported jaundice before voxelotor treatment (Table 3). Patients noticed changes within 24 hours of starting voxelotor (1/18; 6%), within a few weeks (2/18; 11%), and within 1 or 2 months (4/18; 22%). The average rating for impact of jaundice on quality of life decreased from 2.9 to 0.5.

“Well, after I started the voxelotor, my jaundice went away within at least two or three days—no, not even two or three days—at least within 24 hours, the next day.” (17-year-old patient with SCD)

Most caregivers noted either partial or complete resolution of their child's jaundice after starting voxelotor (Table 3).

“No more jaundice, no more yellowing in [their] eyes.” (Caregiver of 9-year-old patient with SCD)

#### 3.5.3. Fatigue

*(1) Fatigue before Voxelotor*. Fatigue was reported by most patients before starting voxelotor: 83% of adults and adolescents (19/23), 80% of caregivers (8/10), and 40% of children (4/10).

“I was just like really tired and I just didn't have a lot of energy. I would come home from school and just immediately go to sleep. I was just exhausted from the littlest things sometimes.” (22-year-old patient with SCD)

The most frequent caregiver-reported impacts of fatigue on their child's everyday life were tiring easily or needing to sleep or nap regularly (4/8; 50%) and being unable to participate in the same activities as other children (3/8; 38%).

“Pretty intense. I mean, [they] can go to school, but [they weren't] able to participate in Running Club anymore. [They aren't] able to participate in PE, really. [They're] just too tired. [They come] home and [nap] in the afternoon after school because [they're] not doing a whole lot after school because [they're] tired. [They complain] that [their] muscles are tired, as well as just [their] body just feeling overly exhausted, but [they take] naps and [sleep] a lot.” (Caregiver of 11-year-old patient with SCD)

One child described how the fatigue or tiredness impacted their ability to play with others and be active.

“Jumping, running, even things like walking or opening the door. Sometimes it's just watching TV, like I can't even watch TV, […] I can't even […] play a game that I really, really like that I really want to play. It's like I just can't.” (11-year-old patient with SCD)

*(2) Improvement in Fatigue with Voxelotor*. Of the adults and adolescents who reported fatigue before starting voxelotor, most reported an improvement in their fatigue symptoms with treatment (Table 3). Positive changes in fatigue were noticed immediately (2/16; 13%), after 3 weeks of taking voxelotor (1/16; 6%), after 1 or 2 months (2/16; 13%), after 2 weeks to 1 month (1/16; 6%), and after participating in activities with family and friends (1/16; 6%).

“I actually wake up rested now, and always living with fatigue I think you forget you're fatigued a lot, so now that I don't have it, my friends are like, ‘I've never seen you this energetic, like what are you doing?' […] My stamina has increased, I can go longer without feeling exhausted.” (22-year-old patient with SCD)

Half of the caregivers reported improvement in their child's fatigue since starting voxelotor treatment (Table 3).

“I mean it was a huge change, and I think the number one, that first thing we saw was that level of fatigue was just gone. [They] learned to ride [their] bike, […] [they] could go swimming, […] [they] could hang with [their] friends all day long. We noticed that almost immediately, and that was the most remarkable change, was that level of fatigue was just gone.” (Caregiver of 10-year-old patient with SCD)

The majority of the children reported an improvement in their fatigue after starting voxelotor (Table 3).

“We went for the walk and it was a one-mile walk, and I was the front of the line.” (10-year-old patient with SCD)

### 3.6. HRQOL

#### 3.6.1. HRQOL before Voxelotor

When asked to discuss their overall HRQOL prior to voxelotor, 57% of adults and adolescents (13/23) described their quality of life as poor.

“I would have to manage everything that I do according to my transfusions, which was every 4 weeks, and then, once I got my transfusion, that would at least give me energy and less pain for at least two and a half weeks. So, really I had two and a half good weeks out of every month before Oxbryta […] your quality of life can't always be so well and so amazing with just two and a half good weeks.” (37-year-old patient with SCD)

Caregivers acknowledged the impact of SCD on their lives, while responses from children were mixed.

“It […] wasn't that bad. […] we have a pretty good life. But definitely at that point being sick and […] in the hospital and not feeling good was a regular part of [their] life for sure. […] We didn't go on vacation without having a hospital plan, we didn't go anywhere where we couldn't get access to care for [them], because […] we have been on numerous vacations where [they've] ended up in the ER.” (Caregiver of 10-year-old patient with SCD)

#### 3.6.2. Improvement in HRQOL with Voxelotor

When asked about their HRQOL, the majority of the adults and adolescents reported that their HRQOL had improved to some extent since starting voxelotor (Table 3). Improvements attributed to voxelotor treatment included reduced crises, more energy, better adherence to the medication, fewer visits to the hospital, and improved social life. The average rating of overall HRQOL improved from 6.1 to 7.7 (range 4-10).

“I really don't even feel like I have sickle cell anymore.” (22-year-old patient with SCD)

Most caregivers reported that their child's HRQOL had improved since starting voxelotor treatment (Table 3).

“Very much improved, very much improved. I think within a month or two of being on the medication, [they were] student of the month, and they [the school] didn't know any of what changes we had done medically. It's just [they] really just started to do so much better.” (Caregiver of 11-year-old patient with SCD)

Out of the 8 children who were asked to describe their HRQOL after initiating voxelotor, most reported feeling generally positive or better than they felt before starting voxelotor (Table 3).

“It's [life is] easier.” (9-year-old patient with SCD)

**Figure 1:**
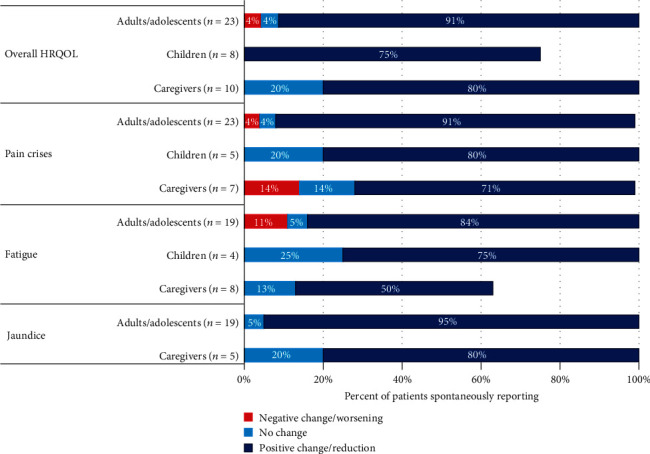
Change in symptoms and overall HRQOL as spontaneously reported by adults and adolescents, children, and caregivers. HRQOL: health-related quality of life.

A summary of the changes in overall HRQOL is presented in Figure 1.

#### 3.6.3. HRQOL in Adults and Adolescents

The adult and adolescent cohort was further asked about the impacts of SCD on specific topics related to HRQOL: everyday activities, social life and relationships, work and school, and emotional health and well-being, which are detailed in the following sections.

*(1) Everyday Activities*. *Baseline before Voxelotor*. Adults and adolescents reported the numerous ways their symptoms impacted their day-to-day lives: inability to get out of bed or do anything (12/23; 52%), general restrictions or limitations on daily activities (7/23; 30%), difficulty with physical activities (6/23; 26%), need for hospitalization (5/23; 22%), and inability to complete chores/household duties (3/23; 13%).

“Everything stops, even my family. Everything stop for them, because there's nothing you can do when you're in a pain crisis, other than be in bed and hopefully take care of yourself. If not, then you'd have to go to the hospital. There is no way you can just push through a pain crisis, just go on with your daily life.” (37-year-old patient with SCD)

*Improvements with Voxelotor*. After treatment with voxelotor, more than half of the adults and adolescents experienced positive changes in their symptoms and symptomatic effects on everyday activities (Table 3). Due to improvements in fatigue, patients reported being generally more able and less tired (8/16; 50%), able to exercise or play sports (4/16; 25%), and able to complete housework and other daily activities without experiencing symptoms (4/16; 25%).

“I'm able to do more, get a lot of things done, I have more energy. I'm able to get up and about and move around more frequently.” (33-year-old patient with SCD)

*(2) Social Life*. *Baseline before Voxelotor*. Most adults and adolescents (17/23; 74%) reported impacts of pain and fatigue on their social life, including cancelling scheduled activities (8/17; 47%), having a limited/nonexistent social life (5/17; 29%), missing out on activities with family/friends (4/17; 24%), and lashing out at or withdrawing from their friends and family (4/17; 24%).

“[…]I miss things or celebrations, sometimes I'd be too sick to go do what I wanted to do, but I always find a way to stay connected with the people I care about. It did affect me with my mental health though. Sometimes I would withdraw from my friends and just kind of turtle to recover from what I went through.” (22-year-old patient with SCD)

The impact of jaundice on social life predominantly involved receiving comments about patients' yellow eyes (9/17; 53%).

“Usually your eyes are the first things that people see […] I always get the comment like ‘oh, are you sick?' Or […] ‘are you an alcoholic?' […] I've had so many crazy things […] said to me, and that doesn't make me emotionally feel great.” (39-year-old patient with SCD

*Improvements with Voxelotor*. Voxelotor treatment improved the social life of more than half of adults and adolescents who were able to generally socialize more (6/13; 46%), socialize or date more (4/13; 31%), spend more time with family (3/13; 23%), and improve social interactions due to reduced or resolved jaundice (3/13; 23%) (Table 3).

“I was able to do more with my friends, and it just made life easier overall.” (14-year-old patient with SCD)

“[…] I guess from the jaundice, that has helped emotionally […] I don't have the fear of like ‘oh, if I meet someone new or that Fed-Ex person comes to drop off my package, I'm going to try to look them in the eye,' like I feel more confident, so that has helped.” (39-year-old patient with SCD)

*(3) Work and School*. *Baseline before Voxelotor*. A total of 74% of adults and adolescents (17/23) reported the impacts of their symptoms on work or school. Impacts on school life included attendance (6/17; 35%), schoolwork or grades (4/17; 24%), and feeling tired during class (3/17; 18%). Work impacts included fewer working hours (3/17; 18%) as well as poor support at work (3/17; 18%).

“[…] You can't do anything because you are in the hospital, so playing catch up when you get out and trying to manage stuff while you're in there.” (38-year-old patient with SCD)

“[…] you only have so many days you can call in sick, […] you try to explain I had to come into work a little late. So that's my, I really have to try to push through.” (39-year-old patient with SCD)

*Improvements with Voxelotor*. One-third of the adults and adolescents reported improvements in work- or school-related impacts after treatment with voxelotor (Table 3).

“I definitely have more time to do my work. I'm not behind anymore. So, there's an improvement there. I'm able to maintain a really good GPA now.” (22-year-old patient with SCD)

“I wouldn't have any problems completing any assignments at my job or anything like that, tiredness or anything of that sort. Like I would get through the day with no problems. […] Fewer disruptions with […] the illness.” (20-year-old patient with SCD)

*(4) Emotional Health and Well-Being*. *Baseline before Voxelotor*. Most adults and adolescents (96%; 22/23) reported emotional or mood-related effects associated with their SCD, including sadness or depression (4/22; 18%). Specifically because of their pain, patients reported feeling sad or depressed (6/22; 27%), frustrated or annoyed (5/22; 23%), and stressed (3/22; 14%).

“[…] I think it started like growing into like a big issue for me, and I didn't want to live with it anymore. And I was just tired of the sickle cell pain and everything, so it impacted me emotionally a lot.” (14-year-old patient with SCD)

*Improvements with Voxelotor*. Overall, most adults and adolescents expressed improvement in their emotional health and well-being after treatment with voxelotor, including generally improved emotional health (3/16; 19%) (Table 3). The average severity rating of emotional impacts decreased from 6.5 to 5, and the average rating for the impact of emotional symptoms on quality of life increased from 5.75 to 6. One patient rated their quality-of-life impact a 10 “in a good way,” which likely skewed the average rating.

“Like knowing that my numbers look better every time I go to the doctor and get blood drawn has definitely made me happier about being on the drug. I don't know if I would say that it directly affected my overall emotional health, but I think just knowing that has definitely made me happier about where my sickle cell is going and how it's going to get better.” (25-year-old patient with SCD)

### 3.7. Pain Medication

All of the adults and adolescents (23/23; 100%) reported the use of some analgesic before starting voxelotor. Since starting voxelotor, 48% of adults and adolescents (11/23) reported reduced reliance on pain medications, including less-frequent use of over-the-counter or prescription pain medication (11/11; 100%), a reduced need for over-the-counter or prescription medications (6/11; 55%), use of over-the-counter medication in lieu of prescription medication (4/11; 36%), and greater relief from existing medications (3/11; 27%). Almost all caregivers (9/10; 90%) reported the use of some analgesic for their child. With voxelotor treatment, more than half of the caregivers (6/10; 60%) described a reduction in how frequently their child required pain medication.

## 4. Discussion

This is the first semistructured interview study reporting the experiences of adults, adolescents, and children treated with voxelotor, a HbS polymerization inhibitor, for SCD. Findings provide information on the symptomatic changes that patients experienced before and after taking voxelotor and how these changes affected their quality of life. Our study complements the growing body of knowledge regarding patient and caregiver quality of life and the emotional and financial burden of SCD.

Findings from the cross-sectional Sickle Cell World Assessment Survey (SWAY) study, conducted in 16 countries and surveying more than 2000 patients with SCD aged 6 to 90 years, demonstrated a high patient-reported symptom/complication burden, substantial unmet needs in care, and negative impact on patient quality of life in SCD [36]. In the study, most patients (94%) were receiving ongoing traditional treatments for SCD, including folic acid, hydroxyurea, opioids, over-the-counter pain medication, and antibiotics. VOCs and fatigue were the most common symptoms reported by patients, and a higher impact of SCD on aspects of daily life, emotional well-being, schooling, and employment was found among patients who reported these symptoms compared with those who did not. Notably, the most commonly selected treatment goal for patients was improving quality of life. SWAY highlighted the need for newer therapies that can target symptoms that are highly prevalent and have the greatest impacts on patients' lives.

In the current study, almost all patients in all age groups reported pain crises before receiving voxelotor. Most patients reported improvements in their pain crises and a reduction in their use of pain medications after receiving voxelotor. Patients and caregivers also reported improvements in jaundice and fatigue. Almost all patients in this study experienced improvements in symptoms and HRQOL after treatment with voxelotor. Further evaluation of HRQOL in the adult and adolescent cohort revealed improvements in everyday activities, social life and relationships, work and school, and emotional health and well-being. These findings highlight the potential benefit of voxelotor beyond improving hemoglobin levels in adults, adolescents, and children with SCD.

Although both children and their caregivers were queried, many of the children interviewed were not descriptive in their responses. This is potentially due to recall issues in children, as many began voxelotor treatment at a young age and could not accurately recall their pretreatment experience. Thus, the inclusion of caregivers was necessary to understand the younger patients' experiences with voxelotor. Although the frequencies of concepts reported by children did not align exactly with those reported by the caregivers, the same patterns emerged, including improvement in symptoms and increased ability to participate in physical and social activities. Additionally, few children mentioned the emotional impacts of their SCD symptoms, whereas most caregivers did discuss these impacts. Due to their young age, children may be less likely to recognize how their symptoms impact their emotional health and well-being. Similarly, none of the children reported symptoms of jaundice, whereas several caregivers identified jaundice as a symptom in their child and reported improvement or resolution of jaundice with voxelotor treatment. Including caregivers in this interview study filled potential knowledge gaps and therefore should be considered when studying experiences with children.

This study included a sufficient sample size for a semistructured interview study, as concept saturation in both cohorts was evident. Since the adult and adolescent cohort was recruited from 4 clinical sites located across different regions of the United States, results from these age groups are likely generalizable; however, the generalizability of the results from the child-caregiver dyads is limited by the small sample size (*n* = 10), nonrandom sample selection, and recruitment from only 1 center. The small sample size for the child-caregiver cohort was the result of voxelotor treatment having not received marketing authorization for patients under 12 years of age at the time of this study's data collection. Thus, recruitment of children and caregivers was limited to 1 clinical center, and all pediatric patients were enrolled in the voxelotor clinical trial at this site.

Because this was a cross-sectional study, self-reported patient data may be limited by recall, especially for young children and those on voxelotor treatment for more than 1 year. The difficulty in recalling symptoms before treatment may make participants less likely to describe a clear benefit of voxelotor treatment. Another limitation includes the retrospective nature of this study and its broad goal to gather data on patient and caregiver experiences.

## 5. Conclusions

This is the first semistructured interview study to report the experiences of adults, adolescents, and children treated with voxelotor, a first-in-class HbS polymerization inhibitor. The results of this study highlight the utility and benefit of voxelotor as a treatment for SCD from the patient perspective. The improvements in symptoms and HRQOL reported by patients and caregivers support the potential of voxelotor beyond increasing Hb. Obtaining qualitative data in the form of patient and caregiver perspectives provides insights regarding the meaningful treatment benefits of voxelotor. These findings should aid in the design of postmarketing surveillance quantitative studies that require SCD-specific patient-reported outcome measures.

## Figures and Tables

**Table 1:** Sociodemographic information for adults and adolescents.

Adult and adolescent characteristic	Sample (*n* = 23); *n* (%)		
Sex			
Male	8 (35)		
Female	15 (65)		
Age (years)			
Mean (SD)	23 (7.4)		
Range	13-39		
Race/ethnicity			
Black/African American	22 (96)		
Latinx	1 (4)		

Highest level of education completed	Total (*n* = 23)	Adolescents (*n* = 7)	Adults (*n* = 16)
Less than high school	6 (26)	6 (26)	0
High school/GED	9 (39)	1 (4)	8 (35)
Some college	2 (9)	0	2 (9)
Bachelor's degree	2 (9)	0	2 (9)
Not answered	4 (17)	0	4 (17)

Employment status	Total (*n* = 23)	Adolescents (*n* = 7)	Adults (*n* = 16)
Unemployed	2 (9)	0	2 (9)
Student	8 (35)	7 (30)	1 (4)
Employed part-time	4 (17)	0	4 (17)
Employed full-time	5 (22)	0	5 (22)
Not answered	4 (17)	0	4 (17)

Time on voxelotor (years)			
Mean (SD)	0.7 (0.47)		
Range	0.25-1.7		

Baseline hemoglobin			
Mean (SD)	8.4 (1.5)		
Range	5.9-12.2		

Recent hemoglobin			
Mean (SD)	9.2 (1.5)		
Range	6.7-11.5		

GED: General Education Diploma; SD: standard deviation.

**Table 2:** Sociodemographic information for children and caregivers.

Child characteristic	Sample (*n* = 10); *n* (%)
Sex	
Male	3 (30)
Female	7 (70)
Age (years)	
Mean (SD)	8.9 (1.6)
Range	6-11
Race/ethnicity	
Black/African American	10 (100)
Latinx	0 (0)
Time on voxelotor (years)	
Mean (SD)	0.98 (0.6)
Range	0.25-2
Baseline hemoglobin	
Mean (range)	9 (7.6-11.1)
Recent hemoglobin	
Mean (SD)	9.6 (1.3)
Range	(8.2-12.2)

Caregiver characteristic	Sample (*n* = 10); *n* (%)
Sex	
Male	3 (30)
Female	7 (70)
Age (years)	
Mean (SD)	32.3 (3.1)
Range	30-38
Race/ethnicity	
Black/African American	10 (100)
Latinx	0 (0)
Highest level of education completed	
High school/GED	8 (80)
Associate degree	0 (0)
Bachelor's degree	1 (10)
Master's degree	1 (10)
Employment status	
Unemployed	1 (10)
Employed full-time	9 (90)

GED: General Education Diploma; SD: standard deviation.

**Table 3:** Improvements in spontaneously reported symptoms and HRQOL per cohort with voxelotor treatment.

Patients reporting improvements, *n*/*N* (%)	Adults and adolescents	Caregivers	Children
Pain crises	21/23 (91.3)	5/7 (71.4)	4/5 (80.0)
Jaundice	18/19 (94.7)	4/5 (80.0)	
Fatigue	16/19 (84.2)	4/8 (50.0)	3/4 (75.0)
Overall HRQOL	21/23 (91.3)	8/10 (80.0)	6/8 (75.0)
Everyday activities	15/23 (65.2)	—	—
Social life	13/23 (56.5)	—	—
Work and school	9/23 (39.1)	—	—
Emotional health and well-being	16/23 (69.6)	—	—

HRQOL: health-related quality of life.

## Data Availability

The data that support the findings of this study are available from the corresponding author upon reasonable request.
